# Narrative Case Notes Have the Potential to Predict Seclusion 3 Days in Advance: A Mixed-Method Analysis

**DOI:** 10.3389/fpsyt.2019.00096

**Published:** 2019-02-28

**Authors:** Clara Stepanow, Jefim Stepanow, Marc Walter, Stefan Borgwardt, Undine E. Lang, Christian G. Huber

**Affiliations:** ^1^Universitäre Psychiatrische Kliniken Basel, Universität Basel, Basel, Switzerland; ^2^Department of Urology, Kantonsspital Baselland, Liestal, Switzerland

**Keywords:** aggression, coercion, emotional involvement, mixed-methods, narrative notes, risk assessment, word count, subjectivity

## Abstract

**Objectives:** Current risk assessment tools can predict problematic behavior and the need for coercive measures, but only with a moderate level of accuracy. The aim of this study was to assess antecedents and triggers of seclusion.

**Methods:** Narrative notes of health care professionals on psychiatric inpatients were analyzed daily starting 3 days prior to seclusion in the case group (*n* = 26) and compared to a matched control group without seclusion (*n* = 26) by use of quantitative and qualitative research methods, based on qualitative content analysis.

**Results:** Quantitative measures showed more aggression in the case group with highly significant differences between the groups (*p* < 0.001) at all measurement times. Seclusion was significantly associated with the total word count of the narrative notes. Subjective emotional expressions by staff were more apparent before seclusion (*p* = 0.003). Most frequently, subjective expressions regarding “arduous/provocative” (*p* < 0.001) and “anxious” (*p* = 0.010) sentiments could be identified in the case group. Description of patients' behavior in the case group included more negatively assessed terms (*p* = 0.001). Moreover, sleep loss, refusing medication, high contact frequency, demanding behavior and denied requests were present in a significantly higher frequency before seclusion. Expressions like “threatening” (*p* = 0.001) were found only before seclusion and appeared to have the function of personal risk assessment. The expression “manageable” (*p* = 0.035) appeared often in difficult situations that could still be handled.

**Conclusion:** Several factors preceding seclusion could be identified. Narrative notes of staff already showed differences 3 days before the escalation. Particularly the word count, the analysis of terms describing patients' behavior, subjective expressions of staff, and terms used as a function of personal risk assessment could help to provide better predictions of aggressive incidents and to prevent coercive measures.

## Introduction

Violence and aggression in mental health care units represent an imminent danger for patients and health care professionals (HCP). In high-income countries, almost one in five acutely admitted psychiatric inpatients commit an act of physical violence (1). Aggression defined as verbal or physical abuse and intimidation (2) should be identified early to prevent further escalation and coercion, which may cause severe psychological distress and is potentially traumatic for both patients (3–5) and staff (6–8).

A number of risk assessment tools to predict violence and reduce severe incidents or the need for coercion are available, but only reach a moderate level of predictive accuracy (9). Correspondingly, a variety of antecedents associated with aggressive behavior have been found in previous investigations (10), encompassing patient symptoms, behavioral and emotional cues, interactional factors (patient-patient or patient-staff interaction), external issues, and structural issues (e.g., environmental factors within wards). In a systematic review and meta-analysis, Dack et al. (11) summarized that the following patient related clinical factors were known to be associated with aggressive behavior: psychiatric in-patients being male, of young age, not married, involuntarily admitted, diagnosed with schizophrenia, having a greater number of previous admissions, a history of violence, self-destruction, and substance abuse. However, patient factors do not entirely explain the variation in the occurrence of dangerous behavior and its management, and a multifactorial cause should be considered instead, also—within others—including staff related factors (11, 12).

Indeed, staff-patient interactions can constitute an important precursor of aggressive incidents (13–15). Perceptions, attitudes, and emotional reactions of staff and patients are considered to affect interactional behavior preceding aggressive incidents (15–19). An example for problematic behavior given by Bowers et al. (20) describes health care professional frustration due to repetitive requests at inappropriate times concerning a trivial item. The type of management that is used to deal with problematic patient behavior influences the interactional response and may lead to an escalation (8, 21–23). Escalation can, e.g., be triggered by a controlling (15, 24, 25), over-confident, and punitive management approach by staff (26–29). In addition, restrictive regiments like limiting patients' freedom or denying patients' requests appear to be an essential antecedent of aggressive behavior (10, 14, 20, 30, 31).

Further, healthcare professionals' emotions and subjective perceptions can contribute to de-escalation and escalation of problematic behavior and could potentially be used to improve risk assessment and prediction. For example, emotions and exposure to patient aggression have been investigated in a cross-sectional questionnaire survey of nurses by Jalil et al. (32), who revealed a positive relationship between nurse-reported anger and exposure to—mostly verbal—patient aggression as well as endorsement of coercive violence interventions. Greater work experience was associated with more tolerance toward aggressive behavior (13), probably due to a better in-depth understanding (20, 33). Furthermore, a less tolerant attitude was related to a higher state of burnout traits like emotional exhaustion, depersonalization and personal accomplishment and an inappropriate management of aggressive incidents (13, 34). In contrast, staff on acute inpatient ward is frequently confronted with challenging patient behavior, which may among others result in a higher tolerance for mild-to-moderate aggression with the possible consequence of missing early warning signs. This might be accompanied by the tendency for underreporting of aggressive incidents (35). In summary, there is evidence for a possible role of staff emotions and subjective experience in the causation and prediction of problematic behavior and coercive measures, but this topic is currently under-researched.

The current study differs from preexisting studies by investigating precursors of seclusion in narrative case notes. Narrative notes contain a wide range of information and were used in previous studies as a source to capture specific information not otherwise available, e.g., patients' problem behavior (12, 19, 20, 36). Natural language processing has the potential power of identifying meaningful predictors in narrative notes (37, 38). In fact, there is evidence supporting this approach for the prediction of suicide from a comprehensive study by McCoy et al. (39). Using computerized natural language processing, the authors were able to investigate 845,417 discharges in a retrospective investigation and could show that cases with the expression of positive sentiments in discharge notes were less likely to attempt suicide.

The aim of the present study was to determine antecedents of seclusion by analyzing narrative notes of HCP in a group of patients with seclusion in comparison to a control group without seclusion in a quantitative and qualitative mixed-methods retrospective case-control study, examining the course of inpatient treatment and staff-patient interaction on a daily basis starting from 3 days prior to seclusion.

## Methods

### Inclusion and Exclusion Criteria

All inpatient cases admitted to the Department of Adult Psychiatry of the Psychiatric University Hospital Basel, Switzerland (Universitäre Psychiatrische Kliniken Basel (UPK), Klinik für Erwachsene, Universität Basel, Switzerland) between October 2014 and May 2016 were eligible for entry in the current study. To be included in the case group, persons had to have undergone seclusion during their inpatient treatment. Cases with < 3 days of inpatient treatment prior to seclusion, or without documentation on one or more of the 3 days prior to seclusion (e.g., because of absconding during this period) were excluded. For patients with repeated hospitalizations with seclusions during the observation period, only the first hospitalization with seclusion compatible with in- and exclusion criteria was entered. During the 20-months observation period, 26 inpatient cases were available for analysis.

For inclusion in the control group, 26 inpatient cases hospitalized during the observation period matched for approximate age (±6 years), gender, and main diagnosis according to ICD-10 chapter V main group were randomly selected. In total, 570 case notes from the documentation of 52 patients were included, *M* = 10.96, *Min* = 4, *Max* = 17, *SD* = 2.4.

### Data Collection and Analysis

The following sociodemographic and clinical data of patients were collected from electronic health records (EHR): gender, age, nationality, main diagnosis according to ICD-10, comorbid substance use disorder (SUD), type of admission (compulsory or voluntary), number of previous hospitalizations, duration of inpatient treatment, marital status, highest educational attainment, and current employment status.

Narrative notes of staff consisting of nursing staff, physicians, psychologists, and other HCP were extracted from the EHR on a daily basis starting from 3 days prior to seclusion (day −3, day −2, and day −1) and at the day of seclusion before escalation (day 0). A day was defined as starting with the first entry of the staff morning shift and ending with the last entry from staff night shift. In cases with insomnia where escalation and seclusion occurred during the early morning hours of day 0, the night prior to seclusion was treated as beginning of day 0. For the control group, the treatment day on which seclusion was performed for the case was chosen as day 0 for the control. For example, if the case was subjected to seclusion on the seventh day of inpatient treatment (day 0 for the case), seventh day of the control was defined as day 0 for the control, and narrative notes of 4 days (days −3 to 0) were extracted. In cases where EHR notes were missing during this episode (e.g., because the patient had absconded), the closest possible day toward day 0 in the case group was chosen with a maximum tolerance of ±3 days.

Whole-text-analysis of narrative notes was repeated several times and performed systematically via qualitative content analysis according to Mayring (40) using a structural method approach and frequency analysis. Categories and codes were defined inductively and deductively applying the qualitative data analysis software MAXQDA12 (VERBI Software GmbH, Berlin, Germany). Qualitative coding methods as described by Saldaña (41) contributed to define categories and codes (e.g., magnitude coding, simultaneous coding, structural coding, descriptive coding, *in-vivo* coding, process coding, concept coding, emotion coding, evaluation coding, hypothesis coding, causation coding, values coding, pattern coding, theme coding). CS performed initial data analysis. A second experienced reviewer (physician), HCP of inpatient wards consisting of nurses, a psychologist, a physician of a psychiatric intensive care unit, and another health care professional experienced in qualitative research supplemented the coding process: comments were applied, codes and themes were discussed, and decision-making was made by consensus. Furthermore, CS and JS rated a subset of 30 narrative notes independently regarding the categories “staff subjectivity,” “sleep behavior,” “demanding behavior,” “compliance,” and “high contact frequency,” since these categories entail a higher risk for rater subjectivity. The average kappa corresponded to 0.86 ranging from substantial to almost perfect interrater reliability (κ = 0.63–1.00) (42) with 23/33 (70%) items having a Kappa > 0.80.

### Quantitative Instruments

In addition to qualitative content analysis, the total number of words in staff documentation and the number of individual notes was calculated on a daily basis for days −3, −2, and −1. Word count and number of notes for day 0 were not assessed as seclusion occurred at different times, and thus the time period and amount of documentation on day 0 prior to seclusion showed considerable variation depending mainly on the time of seclusion, but not necessarily on differences in staff documentation. Moreover, the most frequently used terms were analyzed regarding the number of patients with at least one occurrence of the term and the number of days with at least one occurrence.

Furthermore, the following quantitative instruments were rated for each observed day: The Modified Overt Aggression Scale (MOAS) (43) comprising the four dimensions *verbal aggression, aggression against property, auto-aggression*, and *physical aggression* with five items per dimension; the Positive and Negative Syndrome Scale–Excited Component (PANSS-EC) (44) with the five items *poor impulse control, tension, hostility, uncooperativeness*, and *excitement*, rated on a 7-point Likert scale; and the Clinical Global Impressions–Severity of Aggression scale (CGI-A) (45–47), a rating for the global assessment of a patient's aggressiveness, ranging from *no aggressive behavior, slight aggressive behavior, moderate aggressive behavior, and severe aggressive behavior* to *aggressive behavior present*. Both the OAS (48) and the PANSS-EC have been validated for retrospective use (45). Concerning the psychometric properties of the instruments used in this study, an acceptable to good internal consistency could be achieved. In this study, ratings using the MOAS reached a Cronbach's alpha of 0.759 at Day −3, of 0.786 at Day −2, and of 0.801 at Day −1. Cronbach's alpha of the PANSS-EC reached 0.821 at Day −3, 0.822 at Day −2, and was 0.831 at Day −1.Cronbach's alpha was not determined for the CGI-A, as it is a single-item rating instrument.

### Statistical Analysis

Descriptive statistics were performed for sociodemographic and clinical characteristics, quantitative measures of patient aggression, staff documentation word count, and frequently used terms. Shapiro-Wilk tests were used to test normality. χ^2^-tests were applied to test group differences for categorical data, *t*-tests were used for normally distributed continuous data differences, and Mann-Whitney-*U*-test was performed for not normally distributed continuous data.

In addition, logistic regression was performed to examine the predictive power of the variables “word count,” “positive subjectivity,” “unpredictable,” “sleep irregularities,” and “manageable” on membership in the case group. *p* < 0.05 was considered significant, and only two-tailed tests were used. Pearson's *r* was calculated as a measure of effect size according to Cohen (49). All calculations were performed using the software IBM SPSS Statistics version 24 (IBM Corporation, Armonk, NY, USA).

## Results

### Sample Description

Sociodemographic and clinical characteristics of the case group, the control group, and the total sample are listed in Table 1.

**Table 1:** Clinical and sociodemographic characteristics.

	**Case group (*****n*** **= 26)**		**Control group (*****n*** **= 26)**		**Total sample (*****n*** **= 52)**		***p*-value**
**GENDER**							1.000^b^
Female	9	(34.6%)	9	(34.6%)	18	(34.6%)	
Male	17	(65.4%)	17	(65.4%)	34	(65.4%)	
Age (M ± SD)	39.7 ± 11.6		41.2 ± 10.9		40.4 ± 11.2		0.652^a^
**NATIONALITY**							0.458^b^
Switzerland	15	(57.7%)	18	(69.2%)	33	(63.5%)	
Other European countries	9	(34.6%)	6	(23.1%)	15	(28.8%)	
African countries	2	(7.7%)	2	(7.7%)	4	(7.7%)	
**MAIN DIAGNOSIS (ICD-10)**							0.613^b^
F0.x	2	(7.7%)	2	(7.7%)	4	(7.7%)	
F1.x	1	(3.8%)	1	(3.8%)	2	(3.8%)	
F2.x	19	(73.1%)	19	(73.1%)	38	(73.2%)	
F3.x	2	(7.7%)	2	(7.7%)	4	(7.7%)	
F4.x	1	(3.8%)	1	(3.8%)	2	(3.8%)	
F6.x	1	(3.8%)	1	(3.8%)	2	(3.8%)	
**COMORBID SUBSTANCE USE DISORDER**							0.491^b^
Current addiction	11	(42.3%)	7	(26.9%)	18	(34.6%)	
Substance abuse	2	(7.7%)	2	(7.7%)	4	(7.7%)	
None	13	(50.0%)	17	(65.4%)	30	(57.7%)	
Type of admission							0.012^b^
Compulsory	16	(61.5%)	7	(26.9%)	23	(44.2%)	
Voluntary	10	(38.5%)	19	(73.1%)	29	(55.8%)	
Number of previous hospitalizations (M ± SD)	11.9 ± 10.7		8.6 ± 9.8		10.3 ± 10.3		0.289^a^
Duration of inpatient treatment (M ± SD)	63.0 ± 59.0		61.4 ± 38.5		62.2 ± 49.4		0.398^a^
**MARITAL STATUS**							0.375^b^
Single	14	(53.8%)	18	(69.2%)	32	(61.5%)	
Married/cohabitating	2	(7.7%)	2	(7.7%)	4	(7.7%)	
Married/living separately	3	(11.5%)	0	(0%)	3	(5.8%)	
Divorced	1	(3.8%)	2	(7.7%)	3	(5.8%)	
Unknown	6	(23.1%)	4	(15.4%)	10	(19.2%)	
**HIGHEST EDUCATIONAL ATTAINMENT**							0.149^b^
Incomplete education	0	(0%)	1	(3.8%)	1	(1.9%)	
Obligatory primary school	11	(42.3%)	4	(15.4%)	15	(28.8%)	
Grammar school	2	(7.7%)	3	(11.5%)	5	(9.6%)	
Apprenticeship	2	(7.7%)	8	(30.8%)	10	(19.2%)	
University/College	2	(7.7%)	2	(7.7%)	4	(7.7%)	
Unknown	9	(34.6%)	8	(30.8%)	17	(32.7%)	
**EMPLOYMENT**							0.530^b^
Unemployed	3	(11.5%)	7	(26.9%)	10	(19.2%)	
Protected employment	5	(19.2%)	2	(7.7%)	7	(13.5%)	
Invalidity/retirement	10	(38.5%)	9	(34.6%)	19	(36.5%)	
In training	2	(7.7%)	3	(11.5%)	5	(9.6%)	
Full time job	1	(3.8%)	0	(0%)	1	(1.9%)	

*Values are given in absolute numbers and percentage or in mean (M) and standard deviation (SD). ICD-10, International Statistical Classification of Diseases and Related Health Problems, 10th revision*.

a *t-test*.

b *χ^2^-test*.

Concerning the criteria used for matching cases and controls, included patients had a mean age of 39.7 (range: 19–64) years, 65.4% (34/52) of the patients were male, and 73.2% (38/52) had a main diagnosis of schizophrenia-spectrum disorder.

In the case group, compulsory admission was more frequent (61.5 vs. 26.9%, *p* = 0.012). Concerning comorbid SUD, more patients with a current addictive disorder (42.3 vs. 26.9%) and fewer patients without SUD (50.0 vs. 65.4%) were present in the case group, but these differences were not statistically significant. In addition, no significant differences emerged concerning the nationality, duration of inpatient treatment, marital status, number of previous hospitalizations, highest educational attainment, and current employment previous to hospitalization.

### Measures of Aggressive Behavior

Patients in the case group showed more aggressive behavior than patients in the control group as assessed with the MOAS, PANSS-EC and CGI-A (cf. Table 2). Between-group differences were highly statistically significant (*p* < 0.001) for each observation day beginning from day −3, with an effect size of *r* = 0.4–0.75 (medium to high effect size). Furthermore, an increase of aggression scores from day −3 to day 0 became evident for the case group for all quantitative measurements.

**Table 2:** Quantitative measures of aggression in the case and control groups over the course of the observation period (days −3 to day 0).

**Group**	**Day**	**MOAS total score**	**PANSS-EC total score**	**CGI-A score**
		**M ± SD**	**M ± SD**	**M ± SD**
Case group (*n* = 26)	Day −3	0.7 ± 1.1	13.9 ± 7.3	1.9 ± 1.3
	Day −2	1.2 ± 1.7	16.7 ± 7.5	2.5 ± 1.7
	Day −1	1.4 ± 1.9	19.4 ± 7.9	2.7 ± 1.6
	Day 0	1.9 ± 2.5	23.7 ± 8.8	3.0 ± 1.7
Control group (*n* = 26)	Day −3	0.0 ± 0.0	6.0 ± 2.0	1.0 ± 0.0
	Day −2	0.0 ± 0.0	7.2 ± 2.7	1.0 ± 0.0
	Day −1	0.0 ± 0.0	6.5 ± 3.2	1.1 ± 0.4
	Day 0	0.0 ± 0.2	7.2 ± 3.9	1.1 ± 0.6

*Total scores of the Modified Overt Aggression Scale (MOAS), Positive and Negative Symptom Scale for Schizophrenia—Excited Component (PANSS-EC), and the Clinical Global Impressions–Aggression Scale (CGI-A) are given as mean (M) and standard deviation (SD). Explorative comparisons between the case and the control group were performed using Mann-Whitney U-tests and total scores in the case group were significantly higher than in the control group for all scales and time points (p ≤ 0.001; effect size r = 0.45–0.75)*.

### Qualitative and Quantitative Analysis of Narrative Notes

Qualitative analysis revealed 112 variables per observational day, and 400 variables in total. According to the topics covered by the extracted variables, the main focus of the analysis was placed on expressions of “staff subjectivity,” “terms describing patients' behavior,” terms associated with “risk assessment” and “sleep behavior,” “demanding behavior,” “requests,” “high contact frequency,” and “non-compliance.” Example quotes can be found in Table 3.

**Table 3:** Example quotes.

**STAFF SUBJECTIVITY**	
Provoked or arduous	“He wasn't sleeping this night, standing in the corridor, walking back and forth. Persecuting and observing us provocatively. Refusing his medication, when not, he secretly disposes of it. Patient seems very amused by that.” (Case 10, Day −3)
Anxious	“He was running back and forth the corridor, we felt threatened and were worried he could become physically violent.” (Case 8, Day 0)
Pejorative	Coded mostly by use of expressions like, he came up with the idea to have every reason to get money from us“ (Case 15, Day 0) or pejorative terms in a less compassionate context, like “snappish” or “hysterical”
Enthusiastic	“The patient is doing some team sport after being asked, he is doing his own program, stays until the end (of the lesson) and was complimented on being calm.” (Case 21, Day −1)
Compassionate	“Visible and perceptible considerable psychological strain” (Case 20, Day 0)
**QUOTATIONS OF DIRECT SPEECH**	
	“Watch out, you won't have any rights then too!” (Case 3, Day 0)
	“Leave me alone, I am not having any discussion with you!” (Case 9, Day −2)
	“Psychopharmaceuticals are bullshit!” (Case 25, Day −2)
	“Curious smell” (Case 14, Day −3)
**SLEEP BEHAVIOR**	
Insomnia	“…patient stayed up all night long…” (Case 13, Day 0)
Sleeps late	“…was able to sleep after 2 a.m.” (Case 8, Day −2)
Early awakening	“…went to bed at about 11 p.m. He came at about 3.40 a.m., made a tea and tried to occupy himself” (Case 23, Day −1)
Sleep discontinuity	“…went to bed at 10 p.m., slept with one interruption” (Case 2, Day −1)
**DEMANDING BEHAVIOR**	
	“Patient shows no frustration tolerance, whenever he is not getting what he wants he is running around the ward and shouting loudly.”(Case 6, Day −3)
	“Has difficulties with the rules on the ward: different requests like bread and milk in the middle of the night, modifications of the menu, new shoes etc. Gets tensed and uncalm and starts to smash doors, kicking the bin and furniture around–appears very aggressive and threatening.” (Case 22, Day −1)
	“Patient comes to health care personnel every half hour and demands different things.” (Case 15, Day −1)
	“Needs a lot of attention and assistance.” (Case 7, Day −2)
Non-compliance	“…the patient is still refusing his medication…” (Case 19, Day −3)
High contact frequency	“getting in contact frequently every half an hour with different demands” (Case 15, Day −1)

### Staff Subjectivity

Documentation of patients contains primarily descriptions of their behavior, but subjective statements by staff related to their own perception of patient behavior were also discernible. Staff statements that were accompanied with staff emotional involvement became apparent in narrative notes due to distinct subjective expressions and style of phrasing. Subjectivity was grouped into statements with positive valence containing “enthusiastic” and “compassionate” sentiments, or with negative valence containing “provoked/arduous,” “anxious,” and “pejorative” sentiments (cf. Table 4).

**Table 4:** Staff subjectivity.

	**Case group (*****n*** **= 26)**			**Control group (*****n*** **= 26)**			***p-value***
	***n***	***Frq***	***M ± SD***	***n***	***Frq***	***M ± SD***	
**SUBJECTIVITY WITH NEGATIVE VALENCE**							
Provoked/arduous	12	20	0.8 ± 1.1	0	0	0	< 0.001^a^
Anxious	6	8	0.3 ± 0.6	0	0	0	0.010^a^
Pejorative	4	5	0.2 ± 0.5	0	0	0	0.057^a^
Total subjectivity with negative valence			1.3 ± 1.5			0	< 0.001^a^
**SUBJECTIVITY WITH POSITIVE VALENCE**							
Enthusiastic	2	2	0.1 ± 0.3	3	4	0.2 ± 0.5	0.506^a^
Compassionate	10	13	0.5 ± 0.7	8	12	0.5 ± 0.8	0.564^a^
Total subjectivity with positive valence			0.6 ± 0.8			0.6 ± 0.9	0.850^a^
**Total sum**			**1.8 ± 1.6**			**0.6 ± 0.9**	**0.003**^**a**^

*Valences are presented as dichotomous variables (sentiment occurs/does not occur), n indicating the number of patients where a specific sentiment occurs at least once during the observed days (days −3 to 0), Frq indicating number of days with at least one occurrence (days −3 to 0) summed up over all patients in the group, and M ± SD representing mean Frq per group (mean number of days where the sentiment occurred at least once for the group)*.

a *Mann-Whitney U-test*.

In total, subjective statements were three times more apparent in the case than in the control group, *M* (case group) = 1.8, *SD* = 1.6; *M* (control group) = 0.6, *SD* = 0.9; *U*_(52)_ = 182.5, *p* = 0.003. Negative valence statements were only apparent in the case group, *M* (case group) = 1.3, *SD* = 1.5; *M* (control group) = 0, *SD* = 0; *U*_(52)_ = 130.0, *p* < 0.001, but there were no significant differences concerning the frequency of positive valence statements that were present in both groups, *M* (case group) = 0.6, *SD* = 0.8; *M* (control group) = 0.6, *SD* = 0.9; *U*_(52)_ = −0.162, *p* = 0.850.

Specifically, statements regarding “provoked/arduous” sentiments, *M* (case group) = 0.8, *SD* = 1.1; *M* (control group) = 0, *SD* = 0; *U* = 182.0, *p* < 0.001, and “anxious” sentiments, *M* (case group) = 0.3, *SD* = 0.6; *M* (control group) = 0, *SD* = 0; *U*_(52)_ = 260.0, *p* = 0.010, were significantly more present in the case group. “Compassionate,” *M* (case group) = 0.5, *SD* = 0.7; *M* (control group) = 0.5, *SD* = 0.8; *U*_(52)_ = 312.0, *p* = 0.564, “pejorative,” *M* (case group) = 0.2, *SD* = 0.5, *M* (control group) = 0, *SD* = 0; *U*_(52)_ = 242, *p* = 0.057, and “enthusiastic” sentiments, *M* (case group) = 0.1, *SD* = 0.3; *M* (control group) = 0.2, *SD* = 0.5; *U*_(52)_ = 269.0, *p* = 0.506, were documented in both groups with no significant differences in frequency.

### Terms Describing Patients' Behavior

In both groups, staff used distinctive terms to describe a patient's current behavior. In total, 26 terms could be identified that appeared at least 8 times during the observation period. Behavioral terms were analyzed by their emotional value (positive, negative, or other) and assessed regarding their potential association with problematic behavior leading to escalation and ultimately seclusion as estimated by the context of their use (cf. Table 5).

**Table 5:** Most frequently used behavioral terms.

	**Case group (*****n*** **= 26)**			**Control group (*****n*** **= 26)**			***p-value***
	***n***	***Frq***	***M ± SD***	***n***	***Frq***	***M ± SD***	
**NEGATIVE VALENCE TERMS**							
Agitated	16	41	1.6 ± 1.5	7	11	0.4 ± 0.8	0.003^b^
Irritable	16	25	1.0 ± 1.0	4	4	0.2 ± 0.6	0.001^b^
Loud/screaming	14	22	0.9 ± 1.0	2	3	0.1 ± 0.4	< 0.001^b^
Obtrusive/pushy	13	20	0.9 ± 1.1	0	0	0	< 0.001^b^
Restless	11	19	0.8 ± 1.1	6	8	0.3 ± 0.5	0.083^b^
Threatening	10	15	0.5 ± 0.7	0	0	0	0.001^b^
Dysphoric	10	15	0.7 ± 1.1	0	0	0	0.001^b^
Insulting/cursing	9	17	0.6 ± 1.0	1	2	0.0 ± 0.2	0.005^b^
Aggressive	7	11	0.4 ± 0.8	0	0	0	0.005^b^
Psychotic	7	11	0.5 ± 0.9	3	5	0.2 ± 0.6	0.162^b^
Bizarre/foolish	7	10	0.4 ± 0.7	0	0	0	0.005^b^
Provocative	8	10	0.4 ± 0.8	1	1	0.0 ± 0.2	0.010^b^
Volatile	6	9	0.4 ± 0.7	2	2	0.1 ± 0.3	0.115^b^
Distracted	5	8	0.3 ± 0.7	2	3	0.1 ± 0.3	0.205^b^
Total terms with negative valence			9.2 ± 6.5			1.5 ± 2.4	< 0.001^b^
**POSITIVE VALENCE TERMS**							
Friendly	19	32	1.4 ± 1.1	20	41	1.5 ± 1.2	0.940^b^
Calm	14	21	1.0 ± 1.0	19	32	1.2 ± 1.0	0.310^b^
Relaxed	7	10	0.4 ± 0.7	11	13	0.5 ± 0.7	0.376^b^
Reachable	5	4	0.2 ± 0.5	9	12	0.5 ± 0.8	0.176^b^
Adequate	1	3	0.1 ± 0.4	9	11	0.5 ± 0.8	0.007^b^
Good mood	3	3	0.1 ± 0.3	6	9	0.3 ± 0.6	0.242^b^
Cooperative	2	3	0.1 ± 0.3	5	9	0.2 ± 0.5	0.654^b^
Organized	0	0	0	4	8	0.3 ± 0.7	0.039^b^
Total terms with positive valence			3.3 ± 2.2			5.0 ± 3.4	0.039^a^
**OTHER TERMS**^**c**^							
Manageable	13	21	0.8 ± 1.1	6	7	0.3 ± 0.5	0.035^b^
Unchanged	0	0	0	10	11	0.4 ± 0.5	< 0.001^b^
Reclusive	6	8	0.3 ± 0.6	12	19	0.8 ± 1.0	0.054^b^
Sad	1	3	0.1 ± 0.4	4	8	0.3 ± 0.7	0.168^b^
Unpredictable^d^	3	3	0.1 ± 0.3	0	0	0	0.077^b^
Total terms with other valence			1.3 ± 1.2			1.7 ± 1.6	0.292^a^
**Total sum**			**13.8 ± 6.7**			**8.2 ± 4.3**	**0.001**^**a**^

*Terms are presented as dichotomous variables (term occurs/does not occur), n indicating the number of patients where a specific term occurs at least once during the observed days (days −3 to 0), Frq indicating number of days with at least one occurrence (days −3 to 0) summed up over all patients in the group, and M ± SD representing mean Frq per group (mean number of days where the term occurred at least once for the group). Only terms with Frq ≥ 8 for at least one patient groups are presented*.

a *t-Test*.

b *Mann-Whitney U-test*.

c *Terms that could not be classified as “negative” or “positive” valence*.

d *“Unpredictable” occurred < 8 times, but appeared often in context of risk assessment before seclusion*.

Staff described patients with behavioral terms significantly more often in the case group than in the control group, *M* (case group) = 13.8, *SD* = 6.7; *M* (control group) = 8.2, *SD* = 4.3; *t*_(52)_ = −3.5, *p* = 0.001. The description of patients' behavior in the case group included significantly more terms with negative valence, and these terms were potentially related to problematic behavior, *M* (case group) = 9.2, *SD* = 6.5; *M* (control group) = 1.5, *SD* = 2.4; *U*_(52)_ = 80.0, *p* < 0.001.

More precisely, terms like “agitated,” “irritable,” “loud/screaming,” “obtrusive,” “restless,” “threatening,” “dysphoric,” “insulting/cursing,” “aggressive,” “bizarre/foolish,” and “provocative” emerged with a significantly higher frequency in the case group. The most frequently represented behavioral term in the case group was “agitated.”

Patients in the control group were significantly more often perceived to be “adequate,” “organized” and “unchanged,” but were described as “psychotic,” “volatile,” “distracted,” “reclusive,” and “sad” in their behavior in equal frequency as in the case group. Positively assessed terms like “friendly,” “calm,” “relaxed,” “reachable,” “good mood,” and “cooperative” appeared frequently in both groups. However, in total, terms with positive sentiments appeared significantly more often in the control group, *M* (case group) = 3.3, *SD* = 2.2; *M* (control group) = 5.0, *SD* = 3.4; *t*_(52)_ = 2.1, *p* = 0.039. The term “friendly” was the most common behavioral term in the control group, but was not present with a significantly higher frequency compared to the case group.

### Risk Assessment

Expressions like “threatening,” *M* (case group) = 0.5, *SD* = 0.7; *M* (control group) = 0, *SD* = 0; *U*_(52)_ = 208.0, *p* = 0.001, or “unpredictable,” *M* (case group) = 0.1, *SD* = 0.3; *M* (control group) = 0, *SD* = 0; *U*_(52)_ = 299.0, *p* = 0.077, were used more often before an aggressive event or escalation in the case group. They appeared to function as a surrogate of personal risk assessment, and can be considered as a personal assessment if seclusion will become necessary. The term “threatening” shows an increased use in the time preceding seclusion. These expressions were not used by staff documenting the course of treatment in the control group.

The term “manageable,” *M* (case group) = 0.8, *SD* = 1.1; *M* (control group) = 0.3, *SD* = 0.5; *U*_(52)_ = 240.0, *p* = 0.035, shows a similar pattern; it is used more often in the case group, particularly in the context of problematic behavior that could still be handled.

### Sleep Behavior

Sleep behavior was coded as sleep irregularities including “insomnia” defined as total loss of sleep, “late onset of sleep” if patients fell asleep later than 24:00, “early awakening” if patients awoke before 5:00, “sleep discontinuity,” and “no irregularities” (cf. Table 6). In the days before seclusion, patients in the case group showed significantly more sleep irregularities, *M* (case group) = 0.8, *SD* = 1.0; *M* (control group) = 0.3, *SD* = 0.5; *U*_(52)_ = 240.5, *p* = 0.036. Moreover, insomnia increased in the case group toward the day of seclusion and was the type of sleep irregularity with the highest frequency in the case group, *M* (case group) = 0.9, *SD* = 1.0; *M* (control group) = 0.2, *SD* = 0.5; *U*_(52)_ = 194.0, *p* = 0.004. In addition, greater latency to sleep onset, *M* (case group) = 0.7, *SD* = 0.7; *M* (control group) = 0.1, *SD* = 0.3; *U*_(52)_ = 164.5, *p* < 0.001, and early awakening were observed and documented more often in the case group, *M* (case group) = 0.5, *SD* = 0.7; *M* (control group) = 0.2, *SD* = 0.4; *U*_(52)_ = 254.0, *p* = 0.047.

**Table 6:** Sleep behavior, high contact frequency, demanding behavior, requests, and non-compliance.

	**Case group (*****n*** **= 26)**			**Control group (*****n*** **= 26)**			***p-value***
	***n***	***Frq***	***M ± SD***	***n***	***Frq***	***M ± SD***	
Sleep behavior							
Insomnia	14	23	0.9 ± 1.0	4	5	0.2 ± 0.5	0.004^a^
Late Onset of sleep after 12 p.m.	16	19	0.7 ± 0.7	3	3	0.1 ± 0.3	< 0.001^a^
Early awakening before 5 a.m.	10	13	0.5 ± 0.7	4	4	0.2 ± 0.4	0.047^a^
Sleep discontinuity	15	20	0.8 ± 0.8	12	22	0.9 ± 1.2	0.707^a^
**Total sleep irregularities during 4 nights (yes/no)**	**55**	**75**	**0.8 ± 1.0**	**23**	**34**	**0.3 ± 0.5**	**0.036^a^**
High contact frequency	17	52	2.0 ± 1.7	5	8	0.3 ± 0.7	< 0.001^a^
Demanding behavior	22	40	1.5 ± 1.1	11	16	0.6 ± 0.9	0.002^a^
Met requests	5	5	0.2 ± 0.4	5	8	0.3 ± 0.7	0.894^a^
Denied requests	17	28	1.1 ± 1.1	5	7	0.3 ± 0.6	0.001^a^
Non-compliance	26	45	1.7 ± 1.3	11	16	0.1 ± 0.4	< 0.001^a^

*Dichotomous variables are presented (term occurs/does not occur), n indicating the number of patients where a specific term occurs at least once during the observed days (days −3 to 0), Frq indicating number of days with at least one occurrence (days −3 to 0) summed up over all patients in the group, and M ± SD representing mean Frq per group (mean number of days where the term occurred at least once for the group)*.

a *Mann-Whitney U-test*.

Sleep discontinuity showed the highest frequency within the control group, but no significant differences in frequency could be observed between the case and the control groups, *M* (case group) = 0.8, *SD* = 0.8; *M* (control group) = 0.9, *SD* = 1.2; *U*_(52)_ = 319.0, *p* = 0.707.

### Other Behavioral Precursors

High contact frequency of patients with staff was observed and documented more often in the case group, *M* (case group) = 2.0, *SD* = 1.7; *M* (control group) = 0.3, *SD* = 0.7; *U*_(52)_ = 152.0, *p* < 0.001. Furthermore, patients were described as more demanding *M* (case group) = 1.5, *SD* = 1.1; *M* (control group) = 0.6, *SD* = 0.9; *U*_(52)_ = 174.5, *p* = 0.002, e.g., by asking repeatedly for something like being allowed to temporarily leave the ward, receiving cigarettes or food at inappropriate times, inappropriate request like being supplied with alcohol or cannabis, or by ringing the bell repeatedly) and refusing medication more often, *M* (case group) = 1.7, *SD* = 1.3; *M* (control group) = 0.1, *SD* = 0.4; *U*_(52)_ = 96.0, *p* < 0.001. Requests were denied more often in the case group, *M* (case group) = 1.1, *SD* = 1.1; *M* (control group) = 0.3, *SD* = 0.6; *U*_(52)_ = 178.5, *p* = 0.001, but there were no significant differences regarding the frequency of requests that were fulfilled between both groups, *M* (case group) = 0.2, *SD* = 0.4; *M* (control group) = 0.3, *SD* = 0.7; *U*_(52)_ = 333.0, *p* = 0.894.

### Text Characteristics

The extracted narrative notes for the total sample of 52 patients had a mean total word count of 529.7 (*SD* = 296.6) words. Furthermore, narrative notes of the case group contained more words per treatment day than the control group, *M* (case group) = 639.8; *SD* = 322.5; *M* (control group) = 419.6; *SD* = 224.1; *U*_(52)_ = 193.5, *p* = 0.008.

The mean number of individual entries of notes per day over all 52 patients was 11.0 (*SD* = 2.4) with no significant differences between the groups, *M* (case group) = 10.4, *SD* = 2.5; *M* (control group) = 11.6, *SD* = 2.2; *U*_(52)_ = 241.5, *p* = 0.074.

Moreover, patients in the case group were cited more often via direct speech, *M* (case group) = 1.0, *SD* = 1.0; *M* (control group) = 0.3, *SD* = 0.7; *U*_(52)_ = 198.5*, p* = 0.003, most commonly to document patient quotes deemed important as exactly as possible (for examples cf. Table 3).

### Prediction of Seclusion

Logistic regression was performed with word count, positive subjectivity, “unpredictable,” sleep irregularities, and “manageable” as potential predictors of seclusion, yielding a highly significant model, χ^2^(5) = 18.340, *p* = 0.003, *n* = 52. Patients with a higher word count were at a 43.2% higher risk to get secluded, OR 1.432 per 100 words, *p* = 0.018, Nagelkerke *R* ^2^ = 0.396 (corresponding to a medium to high effect size). None of the other included variables emerged as significant predictors of seclusion in this multivariate analysis.

## Discussion

The current case-control study used a mixed methodological approach in order to examine whether precursors of seclusion can be identified in the narrative notes about acutely ill psychiatric inpatients as written by HCP, particularly with regard to subjectivity and emotional involvement. Various significant differences in staff documentation were identified between the groups. Thus, the current study found evidence supporting the hypothesis that an upcoming escalation is preceded by specific characteristics of staff narrative notes in the days before seclusion.

First, seclusion was significantly associated with higher word count in the current study. Notes in the case group were longer and more substantial, while the frequency of staff documentation entries per day showed no significant difference. In particular, a greater extent of behavioral terms and the use of direct speech were prominent in the staff notes describing patients before seclusion. It seems that staff describes problematic behavior more extensively and with different terms, potentially to improve information transfer between staff members working different shifts, to improve justification for upcoming coercive measures, and to ensure legal protection.

Secondly, seclusion was observed to be associated strongly with staff emotional involvement as measured by use of subjective expressions. Results also indicate that emotional involvement in the shape of “provocative/arduous” and “anxious” terms precede impending seclusion. This is remarkable, as staff documentation tends to minimize emotional content to enhance standardization, objectivity, professionalism, and appropriateness for use in potential lawsuits (50). Thus, it can be assumed that only a fraction of what an individual staff member was actually feeling or intending is documented in the their notes. Nevertheless, relevant differences concerning emotional involvement and subjective expressions remain in the documentation on psychiatric intensive care units. It remains unclear if emotional involvement is only associated with problematic behavior or if it also contributes an escalation process leading to seclusion. Although there is a variety of possible underlying causes and triggers, there is evidence that staff emotions may contribute to seclusion. DeBenedictis et al. (51), e.g., identified a negative working climate, and especially anger and aggression among staff members, as resulting in a higher use of seclusion and restraint. In addition, the question rises if staff with a higher susceptibility for provocation or anxiety may be more likely to apply coercive measures. In-line with these considerations, paying greater attention to staff-patient interactions could help increasing awareness for unconscious and emotional processes (16) involved in escalation processes. Staff training in noticing their emotional state early and emphasizing patients' perception might constitute an important part of de-escalation trainings and help prevent coercive measures like seclusion (6, 14). In any case, emotional involvement in narrative case notes could serve as an early warning sign of an impending seclusion.

Altogether, subjective expressions were less prominent in narrative notes than expected. The style of describing patients was widely consistent and personal stylistic features in documentation were rarely notable. Based on our experience, attempts to be as objective as possible and to use a pragmatic language are widespread in hospital routine documentation in German speaking European countries. Sentiments and emotions are normally omitted when standardized documentation is disseminated due to legal and time management reasons. Hamilton et al. (50) described a patient's file as a “domain of management,” where HCP “experience some compelling pressure to adopt, audit and report on their risk management strategies” (p. 90). The presented data raise the question if—in contrary to the current standards—there should be a regular place for subjectivity in psychiatric routine documentation, as it may represent an essential contribution to clinical assessment and may be helpful for the early detection of seclusion.

Third, as assumed, patient behavior preceding seclusion is significantly more often associated with negatively assessed terms like “agitated,” “irritable,” and “loud/screaming.” Similar findings could be shown in a large study by Cullen et al. (36) were “restraint” and “shouting” showed the strongest likelihood of seclusion among all behavioral keywords. Although patients of the present study were equally perceived as “friendly” in both groups, there were—overall—more positively assessed terms in the control group. Patients in the control group were more often described as being “adequate” and “organized,” and their behavioral amplitude was perceived to be less dynamic, described by the term “unchanged.”

Fourth, patients before seclusion had a considerably higher frequency of insomnia. Sleep irregularities are a well-known comorbidity of patients with psychiatric diagnoses and amongst others associated with worsening of symptom severity in schizophrenia (52–54) and an increase in mania symptoms (55, 56). Consistent with previous studies, there is evidence for an association of poor sleep and aggressive behavior in psychiatric patients (57, 58). Despite this evidence for an association between sleep irregularities and coercive measures, which is corroborated by the current study's results, sleep disturbance has received little attention in previous studies analyzing antecedents of seclusion. In addition, patients in the case group were observed as more demanding and refused medication more often compared to patients in the control group. Staff denying patients' requests was present more often before seclusion, which is consistent with previous studies describing restrictions as a frequent antecedent of aggressive incidents (10, 20).

Fifth, specific terms appeared to serve as a personal evaluation of critical situations and personal risk assessment. “Manageable” appeared significantly more often in the case group, which may seem counterintuitive at first. However, “manageable” was applied in the context of problematic behavior that could still be handled, denoting that despite problematic behavior, staff did not deem coercive measures necessary at this time. Terms like “threatening” were exclusively used in the case group and in the context of seclusion. This is in line with the literature. In a study by Foster et al. (8), the main consequence of aggressive incidents was that staff members were feeling threatened. While not necessarily being subjected to actual threatening behavior, they seemed to feel a “threat of what might happen” (8).

The current study stands out due to the combination of quantitative and qualitative methods (59, 60). One of the strengths of a qualitative approach was the opportunity to determine unknown factors preceding seclusion. By including narrative notes not only of nursing staff but of all HCP, generalizability could be enhanced. Validity could be enhanced by independently performing assessments through two researchers with high interrater reliability, comparison with a control group, and inclusion of quantitative assessments. Matching the patients in a case-control study resulted in more homogeneous groups and improved comparability.

However, there are several notable limitations. Although interrater reliability has been assessed, the influence of subjectivity cannot be eliminated in qualitative analysis (61). Since the study evaluated notes written in German, it is unclear if the results can be generalized to settings with other languages or different cultural backgrounds. Furthermore, only patients with a minimum inpatient treatment duration of 3 days prior to seclusion could be considered, although seclusion already can occur in the first days of treatment with one third of aggressive incidents happening in the first seven days of admission (35). In addition, absconded patients could not be included in the analyses, as the study was focused on evaluating narrative notes in the days before seclusion, and assessments could not be performed when text entries were missing. Although the examined patient groups differ with regard to seclusion, they–indeed–also showed a different level of aggression. Therefore, the examined case note characteristics are not attributable to a development leading to seclusion independently of aggression, and the influence of seclusion and aggression cannot be disentangled with the current approach. In- and exclusion criteria therefore introduced a potential selection bias. Furthermore, the study only used retrospectively available data, leading to several limitations, e.g., the underreporting of aggressive events (35) and of subsequent interventions. In addition, although the current study's sample size is adequate for qualitative assessment (62), further replication studies with larger samples are needed.

## Conclusions

Despite professionalism and self-imposed reduction of emotional and subjective content, narrative notes of healthcare professionals still contain normally unused information that is associated with coercive measures like seclusion as early as 3 days before these events. This information might improve risk assessment as well as the early prediction and intervention of aggressive incidents.

Furthermore, the present study indicates that narrative notes should not be completely superseded by fully standardized documentation. Moreover, integrating subjectivity and emotional content in a way compatible with current standards could improve the clinical usability of routine documentation. Integration of a subjective perspective could be technically implemented by introducing an extra text-field dedicated to subjective perception, countertransference, or more subjective personal remarks (e.g., “I was very scared, my colleague hid in the corner of the room.”) into routine electronic documentation. This would probably not lead to a relevant increase in documentation efforts, since the writing process is performed intuitively and without being censored. Text-analysis and evaluation of the risk level would be performed automatically after documentation is finalized. This is, e.g., achievable by generating computerized algorithms for subjectivity and using machine learning on the basis of natural language processing. This approach also allows to include data from structured risk-assessment instruments into the analysis. Apart from the acquisition costs of an appropriate software solution, no additional financial burden is to be expected, as narrative notes are immediately available for the software in most current documentation systems.

In the present study, factors not routinely used for risk assessment like subjective statements, sleep disturbance, and narrative note word count were associated with seclusion. If these results can be replicated and remain valid especially in a prospective setting, e.g., word count could become an economical and simple predictor for escalation. The implementation of automated text analysis would enable routine use of narrative notes in the early detection and prevention of violence and coercion, and this has to be examined in future studies.

## Data Availability

The raw data supporting the conclusions of this manuscript will be made available by the authors, without undue reservation, to any qualified researcher.

## Author Contributions

CS and CH designed the study, analyzed, and interpreted the data. CS and JS collected the data. CS wrote the initial draft of the manuscript. CS had full access to all the data in the study and takes responsibility for the integrity of the data, and the accuracy of data analysis. CS, JS, MW, SB, UL, and CH revised the article critically for important intellectual content and approved the final version of the manuscript.

### Conflict of Interest Statement

The authors declare that the research was conducted in the absence of any commercial or financial relationships that could be construed as a potential conflict of interest.
